# Pain Relief, Functional Recovery, and Chondroprotective Effects of *Angelica gigas* Nakai in Osteoarthritis Due to Its Anti-Inflammatory Property: An In Vitro and In Vivo Study

**DOI:** 10.3390/nu16152435

**Published:** 2024-07-26

**Authors:** Hee-Geun Jo, Chae Yun Baek, Yeseul Hwang, Eunhye Baek, Ho Sueb Song, Donghun Lee

**Affiliations:** 1 Department of Herbal Pharmacology, College of Korean Medicine, Gachon University, 1342 Seongnamdae-ro, Sujeong-gu, Seongnam-si 13120, Republic of Korea; jho3366@hanmail.net (H.-G.J.);cyning20@gachon.ac.kr (C.Y.B.);; 2 Naturalis Inc., 6, Daewangpangyo-ro, Bundang-gu, Seongnam-si 13549, Republic of Korea; 3 RexSoft Inc., 1 Gwanak-ro, Gwanak-gu, Seoul 08826, Republic of Korea; 4 Department of Acupuncture & Moxibustion Medicine, College of Korean Medicine, Gachon University, 1342 Seongnamdae-ro, Sujeong-gu, Seongnam-si 13120, Republic of Korea

**Keywords:** East Asian herbal medicine, osteoarthritis, *Angelica gigas* Nakai, anti-inflammatory, analgesic, chondroprotective

## Abstract

Osteoarthritis (OA), characterized by chronic pain and joint degradation, is a progressive joint disease primarily induced by age-related systemic inflammation. *Angelica gigas* Nakai (AG), a medicinal plant widely used in East Asia, exhibits promising results for such conditions. This study aimed to evaluate the potential of AG as a drug candidate for modulating the multifaceted pathology of OA based on its anti-inflammatory properties. We evaluated the efficacy of AG in pain relief, functional improvement, and cartilage erosion delay using monosodium iodoacetate-induced OA rats and acetic acid-induced writhing mice, along with its anti-inflammatory effects on multiple targets in the serum and cartilage of in vivo models and lipopolysaccharide-stimulated RAW 264.7 cells. In vivo experiments demonstrated significant analgesic and chondroprotective effects of AG, along with functional recovery, in model animals compared with the active controls. AG dose-dependently modulated inflammatory OA pathology-related targets, including interleukin-1β, tumor necrosis factor-α, matrix metalloproteinase-13, and cyclooxygenase-2, both in vitro and in vivo. In conclusion, AG could be a potential drug candidate for modulating the multifaceted pathology of OA. Nevertheless, further comprehensive investigations, involving a broader range of compounds, pathologies, and mechanisms, are warranted to validate these findings.

## 1. Introduction

Osteoarthritis (OA) is a common musculoskeletal disease that affects millions of people worldwide as the population ages [1]. The cartilage and periarticular tissues progressively develop defects with increasing age and undergo repeated degenerative changes, ultimately leading to OA [2]. OA is characterized by chronic pain, progressive joint destruction, and irreversible dysfunction and is increasingly recognized as a global health concern [3]. As predicted by the World Health Organization, OA may emerge as a leading cause of disability and rising healthcare costs by 2030 [4]. Nevertheless, the pathogenesis of OA remains unclear. Notably, the accumulation of biomechanical damage to the joint structures causing anatomical and functional joint deterioration was the widely accepted hypothesis until recently. However, increasing evidence indicates that low-grade synovial and systemic inflammation directly contributes to the progressive pathology of OA [5]. In particular, age-related inflammation mediated by the accumulation of intra-articular senescent cells has recently been proposed as an important pathogenic factor in OA [6,7,8]. Therefore, investigations on candidates targeting systemic low-intensity inflammation and senescent cell inhibition are imperative to developing novel therapeutics to inhibit the progressive pathology of OA.

Owing to the lack of understanding of the highly complex and multifactorial pathophysiology of OA, no currently available drugs can fundamentally modulate the disease beyond symptomatic therapy [9]. Consequently, major clinical guidelines prioritize non-drug therapies, such as weight loss, exercise, and physical therapy, in the management of OA [1,10]. Glucocorticoids and non-steroidal anti-inflammatory drugs (NSAIDs) are widely used as established pharmacotherapies for symptomatic relief of OA. However, NSAIDs may exhibit several long-term adverse events, including increased cardiovascular risk. Furthermore, glucocorticoids remain controversial, with high-quality trials failing to demonstrate their benefits over physical therapy [11,12]. Therefore, OA therapies with improved efficacy and long-term safety are urgently needed. To this end, a novel class of drugs called disease-modifying OA drugs (DMOADs) is being actively investigated, and promising compounds that may inhibit the diverse pathology of OA have been discovered [13]. However, to date, no DMOAD has demonstrated efficacy in large-scale clinical trials or reached the market. Therefore, further research is warranted to investigate additional candidates from different perspectives and simultaneously elucidate their mechanisms.

The search for DMOAD candidates has recently emphasized their ability to modulate multiple targets of different inflammatory mediators. Medicinal plants with a long history of human use are promising candidates because of their unique multi-component anti-inflammatory mechanisms [14,15,16]. East Asian herbal medicine (EAHM) has a comparative safety advantage and has long been used by many people in the region, making it an excellent resource for the discovery of DMOADs, particularly for OA, a disease with high prevalence [17,18,19,20,21,22,23]. Angelicae Radix is an EAHM with extensive clinical application in the treatment of various painful musculoskeletal disorders. This medicinal plant is known as *Angelica gigas* Nakai, *A. acutiloba* (Siebold and Zucc.) Kitag., and *A. sinensis* (Oliv.) Diels in the pharmacopoeias of Korea, China, and Japan, respectively. Notably, numerous modern studies have reported the efficacy of *A. gigas* Nakai (hereinafter referred to as AG), used as Korean Angelicae Radix, against stroke, depression, sleep disorders, epilepsy, inflammatory bowel disease, osteoporosis, metabolic disorders, and other inflammatory conditions, with minimal safety concerns [24]. However, despite its promising pharmacological potential, studies on the therapeutic applicability of AG in inhibiting the progressive pathology of OA remain insufficient.

This study aimed to determine the potential of AG, as a DMOAD candidate, in inhibiting the progressive pathology of OA through its multi-component, multi-targeted anti-inflammatory pharmacology. We evaluated the anti-inflammatory effects of AG on multiple OA-related targets in an in vitro model. We also evaluated its ability to control the multifaceted pathology of OA, including pain, cartilage destruction, and functional decline, in an in vivo model.

## 2. Materials and Methods

All experiments in the present study were performed in accordance with the ARRIVE 2.0 guidelines [25].

### 2.1. Preparation of AG Extract

The dried root of AG used in this study was acquired from Yaksudang Pharmaceutical Co., Ltd. (Seoul, Republic of Korea). It was deposited as voucher specimen (No. D200915014) and verified by Professor Donghun from the Department of Herbal Medicine, College of Korean Medicine, Gachon University in Korea. AG roots (10 g) were ground to powdered form and subjected to reflux extraction at 85 °C for 3 h using 100 mL of 30% ethanol. The extract was then filtered using a 90-mm ADVANTEC filter paper (ADVANTEC Ltd., Chiba, Japan) and then concentrated under decreased pressure at 60 rpm and 40 °C using a Heidolph Laborota 4000 efficient (Heidolph Instruments GmbH & Co., Schwabach, Germany) and freeze-dried at −80 °C using the EYELA FDU-2100 series (SUNILEYELA Co., Ltd., Seongnam-si, Republic of Korea). The sample yield was 9.62%. Powdered AG extracts were dissolved in distilled water for all experiments.

### 2.2. High-Performance Liquid Chromatography (HPLC)

The component analysis of AG was performed through HPLC (1100 series; Agilent, Santa Clara, CA, USA). Decursin and decursinol angelates in AG were analyzed using HPLC-UV (DAD), a C18 column (250 × 4.6 mm, 5 μm), and DW (A) and ACN (B) as mobile phases. Next, 1 mL of MeOH was added to 10 mg of AG and sonicated for 10 min. The decursin and decursinol angelates were separated using the gradient conditions listed in Table 1.

### 2.3. Animal

Male Sprague–Dawley (SD) rats (190–210 g, n = 45) and male ICR mice (30–40 g, n = 40) provided by DBL, Inc. were used to establish OA writhing test models. The animals were housed in an animal facility at 20–24 °C temperature, 40–45% humidity, and a 12-h dark/light cycle, and were acclimatized for 7 days. The rats and mice had ad libitum access to food and water. Animal experiments were accomplished depending on the regulations of the Animal Care and Use Policy of Gachon University (GU1-2022-IA0071-01).

### 2.4. Preparation of Monoiodo-Acetate (MIA) Injection and Diet

This experiment was performed to design an OA model using MIA. The rats were classified into five groups (n = 9 each): sham, control (CON), indomethacin (INDO 3; Sigma, Burbank, CA, USA), AG 100, and AG 300. The AIN-93G diet (Saeronbio Inc., Korea) was administered to the sham and CON groups. The INDO 3 group was fed the AIN-93G diet with indomethacin (3 mg/kg), whereas the AG groups were provided the AIN-93G diet with AG extract (100 and 300 mg/kg). All rats were sacrificed 24 days after MIA injection, and blood and right knee joint cartilage tissue were collected. At the end of the test, all animals were euthanized using CO_2_. The OA model designs are presented in Table 2.

### 2.5. Weight Bearing on the Hind Limb

The OA-induced right hind limb was recorded using an incapacitance meter (IITC LifeScience Inc., Woodland Hills, CA, USA) at 0–24 days after OA induction in SD rats. The average weight balance of each limb was analyzed using the following formula:

Weight-bearing ratio (%) = (weight on right hind limb/weight on left and right hind limbs) × 100.

### 2.6. Cartilage Degradation

The OA-induced rats were sacrificed after 24 days, and the right knees were photographed using a Sony α6600 digital camera (Sony Group Corp., Tokyo, Japan), and the degree of erosion of the arthritic bone was assessed based on macroscopic scoring (Table 3).

### 2.7. Serum Concention Analysis of MIA Rats

Whole blood was extracted after sacrificing the OA rats. Whole blood was centrifuged (10 min, 4000 rpm), and serum was isolated. Multiplex analysis of serum cytokines, including tumor necrosis factor (TNF)-α and interleukin (IL)-1β, was performed using the MultiAnalyte Premixed Kit (R&D Systems Inc., Minneapolis, MN, USA), and their levels were measured using a Luminex analyzer (Luminex Co., Austin, TX, USA). All analyses were performed following the manufacturer’s instructions.

### 2.8. Writhing Test

ICR mice were classified into four groups (n = 8 each) and treated with AG (200 and 600 mg/kg), ibuprofen (200 mg/kg; IBU 200; Sigma, USA), or DW (CON). Ibuprofen was provided as a positive control. First, the mice were orally administered the samples and then injected intraperitoneally with 0.7% acetic acid (10 mL/kg) 30 min after sample treatment. The writhing reaction was analyzed after 10 min. The mice exhibited a twisting response, and the response was assessed for 10 min.

### 2.9. RAW264.7 Cell Culture

The Korean Cell Line Bank (Seoul, Republic of Korea) provided the RAW264.7 cells. The cells were cultured in DMEM containing 10% FBS and 5% P/S at 37 °C and 5% CO_2_ (Gibco, Billings, MT, USA).

### 2.10. Cell Toxicity Measurement and Nitric Oxide (NO) Generation

RAW264.7 cells were cultured with AG (10–300 µg/mL) and lipopolysaccharide (LPS; 500 ng/mL) for 24 h in 37 °C and 5% CO_2_. Cell viability was recorded using the EzCytox (DoGenBio, Seoul, Republic of Korea) following the manufacturer’s instructions. NO concentration was measured at a wavelength of 540 nm. This experiment was performed in triplicate.

### 2.11. Quantitative Real-Time Polymerase Chain Reaction

RNA was isolated from the knee cartilage of OA rats and LPS-treated RAW264.7 cells using the RNA Prep Kit (Bioneer, Daejeon, Republic of Korea). The extracted RNA was reverse transcribed to cDNA using Convert Mix (Bioneer) following the manufacturer’s instructions. The primer sequences are presented in Table 4 and Table 5.

### 2.12. Protein Expression Analysis

OA-induced rat knee cartilage tissues and LPS-treated RAW264.7 cells were loaded with protease inhibitor (Sigma) and radioimmunoprecipitation assay buffer (CST Inc., Danvers, MA, USA), and proteins were extracted using a homogenizer (Benchmark, D1000-E; Tempe, AZ, USA). The proteins were then subjected to BCA analysis (Thermo Fisher Scientific Ltd., Waltham, MA, USA), and equal volumes of proteins were then separated using SDS–PAGE. The separated proteins were transferred onto membranes using a transfer machine (Bio-Rad Laboratories, Inc., Hercules, CA USA) at 15 V for 40 min. Membranes were then blocked using a blocking solution (Bio-Rad) for 15 min and incubated for 24 h at 4 °C with primary antibodies against MMP, IL-1β, IL-6, TNF-α, COX-2, NOS2, and GAPDH to prevent non-specific binding. All the antibodies were purchased from Proteintech Inc., (Rosemont, IL, USA), Cell Signaling Technology Inc., (Danvers, MA, USA), or Abcam Corp. (Danvers, MA, USA). The membranes were then washed with TBST buffer (Bio-Rad) and incubated with secondary antibodies for 1 h at 20–25 °C, followed by treatment with ECL solution (Bio-Rad). The membranes were probed using chemidoc (Azure Bio-systems, Dublin, CA, USA), and the experiment was repeated thrice.

### 2.13. Statistical Analysis

Statistical analyses (one-way ANOVA and Dunnett’s post hoc test) were performed using GraphPad Prism version 9.0 (GraphPad Software, Boston, MA, USA). Data were presented as the mean standard error of the mean, and significance was shown at *p* values.

## 3. Results

### 3.1. HPLC Analysis

**Figure 1:**
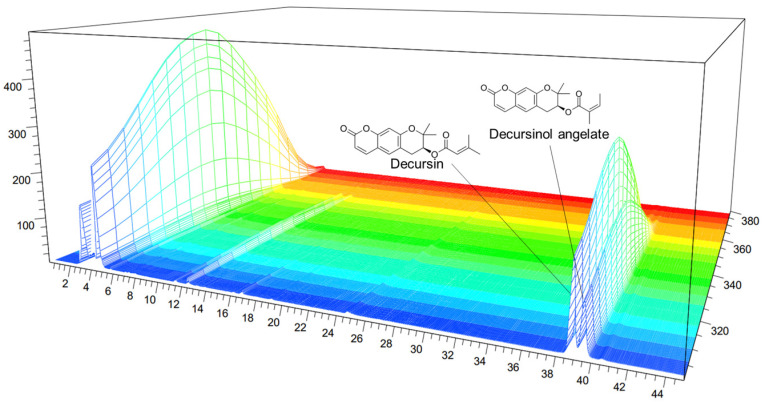
High-performance liquid chromatography (HPLC) chromatogram of the *Angelica gigas* Nakai (AG) extract at 330 nm: decursin and decursinol angelates retention time = 38.72 and 39.388 min, respectively. x-axis: retention time; y-axis: absorbance unit.

The decursinol and decursin angelates in AG were 11.0386 and 5.6396 mg/kg d.m.^−1^, respectively. Figure 1 shows the analytical UV spectra (330 nm) of decursin and decursinol angelates. As decursin and decursinol angelate are structural isomers with the same molecular formula (C_19_H_20_O_5_) and molecular weight (328 nm), they were analyzed in the same wavelength band.

### 3.2. Evaluation of the Analgesic Effects Using MIA Animal Models

**Figure 2:**
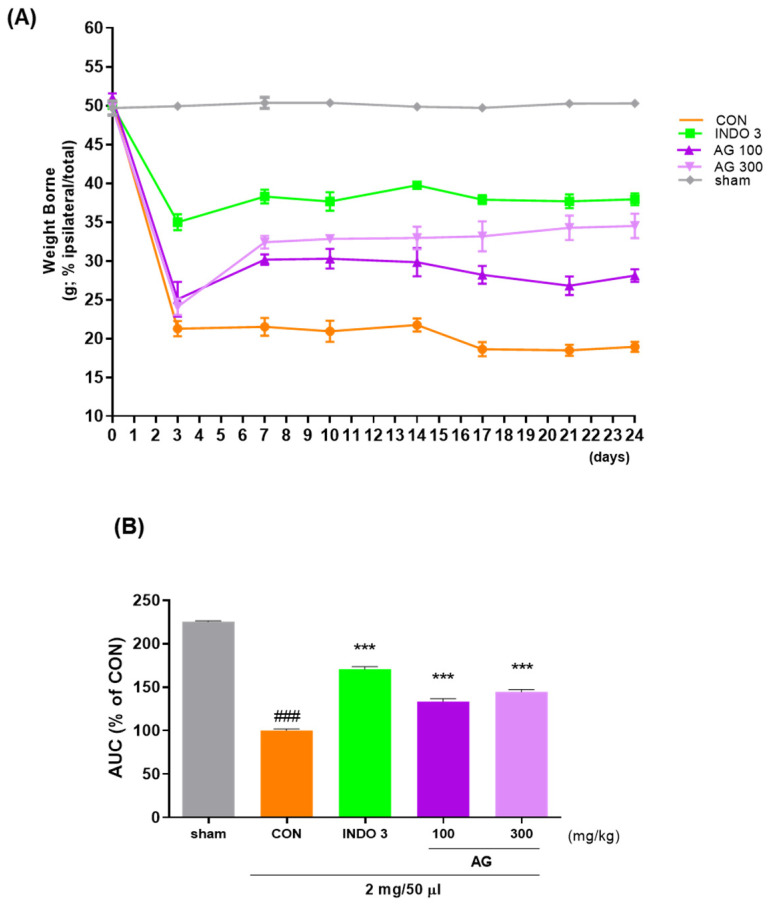
Effect of AG on improving hind limb weight bearing in the osteoarthritis (OA) model; (**A**) the weight bearing of MIA rats in the AG 100, AG 300, and INDO 3 treatment groups for 24 days; (**B**) the AUC was measured from an incapacitance meter. ### *p* < 0.001 vs. sham, *** *p* < 0.001 vs. CON using one-way analysis and Dunnett’s test. AUC: area under the curve; CON: control; INDO 3: indomethacin 3 mg/kg; MIA: monosodium iodoacetate.

Weight bearing capacity on the hind leg was recorded by an incapacitance meter to estimate discomfort and analgesia caused by weight bearing on the hind leg to determine whether OA-induced pain was improved following treatment. The weight bearing capacities of the right and left limbs were measured for 24 days. Notably, the weight-bearing ratio in the CON group was significantly reduced after 10 days, and similar effects to those of INDO-3 were observed thereafter, especially in the AG 300 group (Figure 2).

### 3.3. Cartilage Degradation in MIA Model

**Figure 3:**
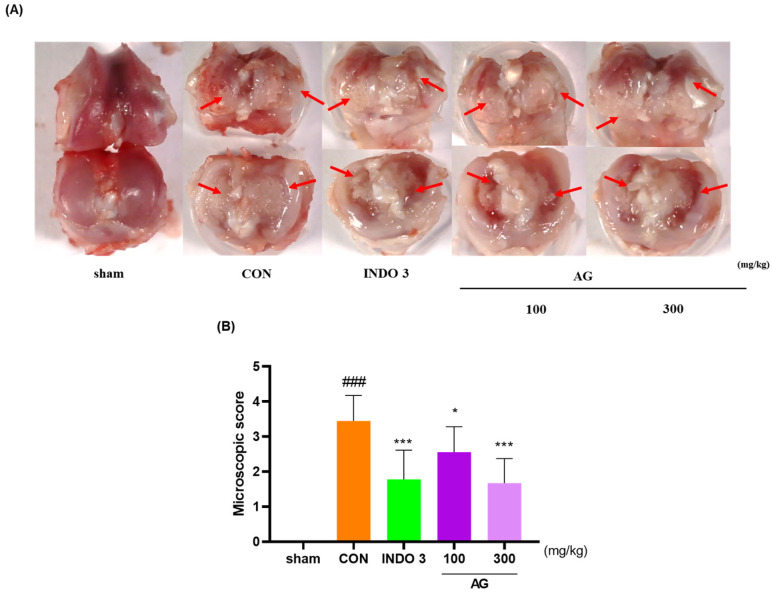
Images of the knee cartilage of OA rats. INDO 3, AG 100, and AG 300 were administered to MIA rats. (**A**) representative photo shown cartilage erosion. Arrows mean the cartilage erosion site. (**B**) Macroscopic score. ### *p* < 0.001 vs. sham, * *p* < 0.05 vs. CON, *** *p* < 0.001 vs. CON using one-way analysis and Dunnett’s test.

The knee joint cartilage of the OA model was sacrificed after 24 days of observation and photographed. Notably, AG could inhibit cartilage damage induced by MIA. The cartilage of the sham group was glossier compared with the cartilage of the CON group (Figure 3A). The surface of the cartilage lost its luster and was rough, along with damage in some areas. According to the macroscopic score, the degree of cartilage degeneration in AG- and INDO 3-treated rats was significantly improved (Figure 3B). In particular, damaged cartilage areas were repaired by AG and INDO 3 to a similar extent.

### 3.4. Serum Pro-Inflammatory Cytokine Levels in MIA Rats

**Figure 4:**
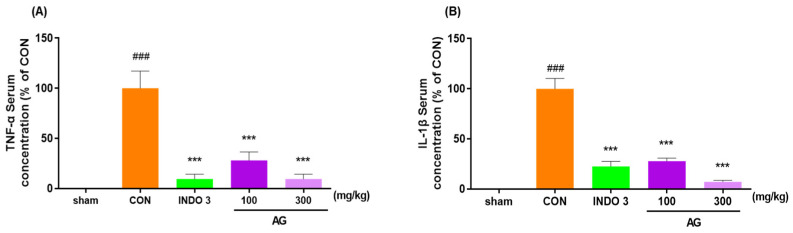
Effects on cytokines in the serum. (**A**) TNF-α and (**B**) IL-1β expression in the serum of MIA rats. Rats were administrated AG 100 and 300 for 24 days. ### *p* < 0.001 vs. sham, *** *p* < 0.001 vs. CON using one-way ANOVA and Dunnett’s test.

The AG group exhibited a significant reduction TNF-α and IL-1β levels in serum compared with the CON group in a dose-dependent manner. Interestingly, AG 300 decreased the TNF-α and IL-1β expressions to lower levels than in the INDO 3 rats (Figure 4).

### 3.5. Analgesic Effects in the Acetic Acid-Induced Pain Animal Models

**Figure 5:**
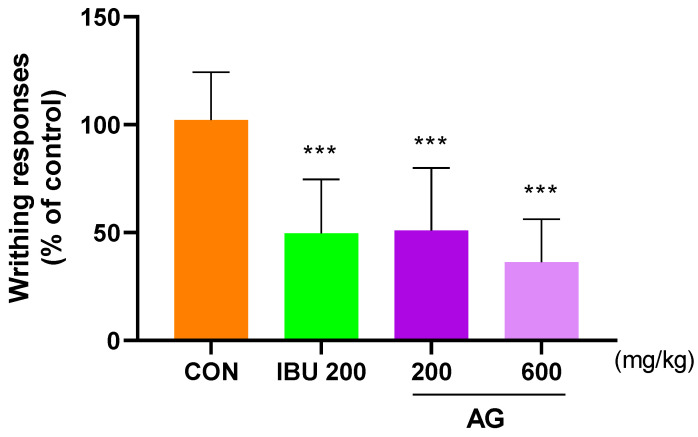
Analgesic effect of AG in OA rats. Mice were treated with IBU 200, AG 200, and AG 600. All mice were intraperitoneally injected with 0.7% acetic acid 10 min prior to the assessment. *** *p* < 0.001 vs. CON using one-way ANOVA and Dunnett’s test.

To determine the degree of improvement in pain, the analgesic effect of AG was investigated using the writhing response with acetic acid. Notably, the writhing response in mice injected with acetic acid was observed after 10 min, and the average value for writhing responses in the CON group was 100. The mice in the IBU 200 and AG 600 groups exhibited average values of 38.73 and 37.53, indicating that AG 600 was more effective than IBU 200 (Figure 5).

### 3.6. Cell Viability and NO Levels in RAW264.7 Cells

**Figure 6:**
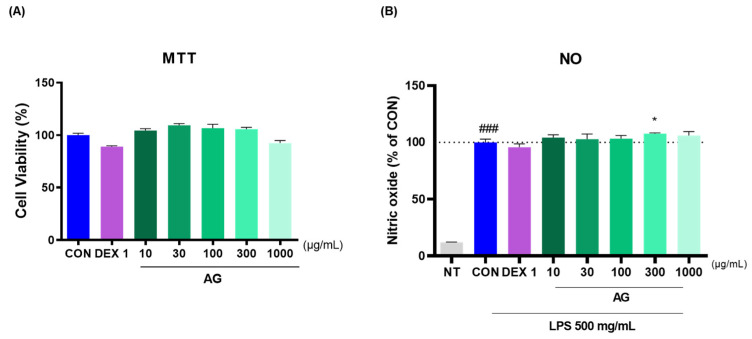
Effects of AG in RAW264.7 cells. (**A**) Cell viability and (**B**) LPS-treated NO generation. ### *p* < 0.001 vs. NT, * *p* < 0.05 vs. CON using one-way ANOVA and Dunnett’s test. DEX: dexamethasone; LPS: lipopolysaccharide; NT: non-treated.

To determine AG toxicity, RAW264.7 cells were treated with AG in a dose-dependent manner, and cytotoxicity was determined using the MTT assay. AG showed no signs of possible cytotoxicity (Figure 6A). To investigate the anti-inflammatory effects of AG, RAW264.7 cells were treated with NO. Notably, AG decreased LPS-induced NO production. No reduction in NO levels was observed in the 300 AG group compared with that in the CON group (Figure 6B).

### 3.7. Anti-Inflammatory Effects of AG in LPS-Treated RAW264.7 Cells

**Figure 7:**
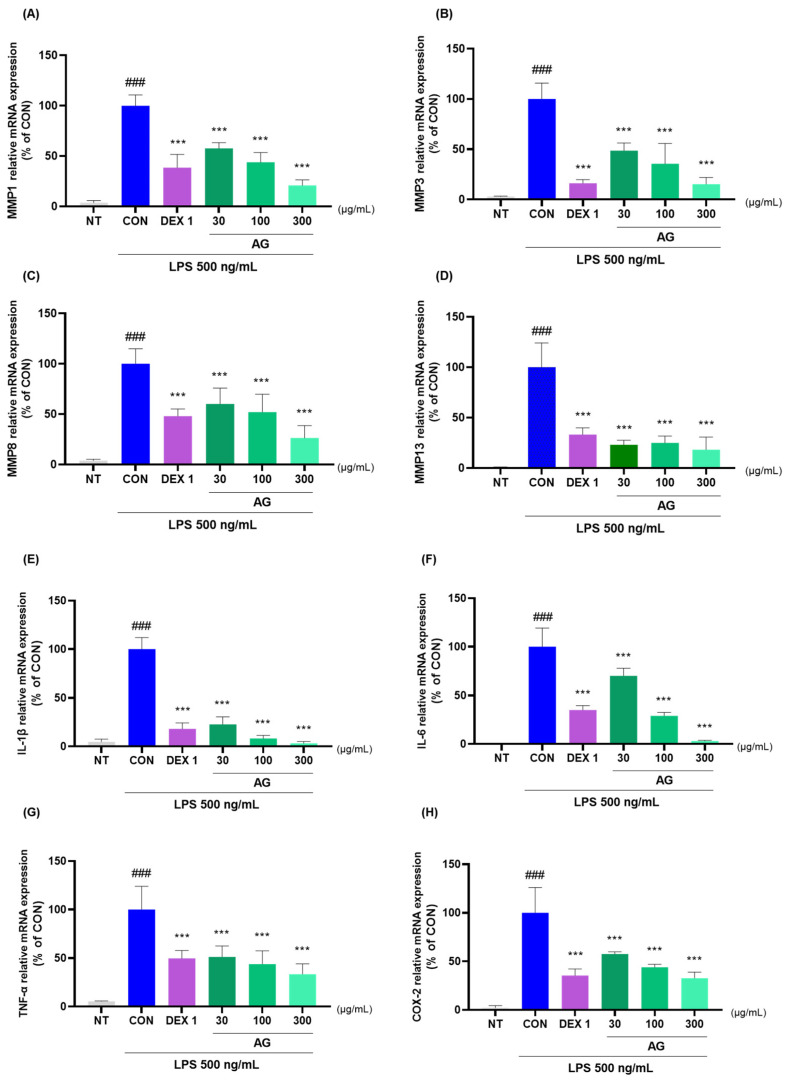

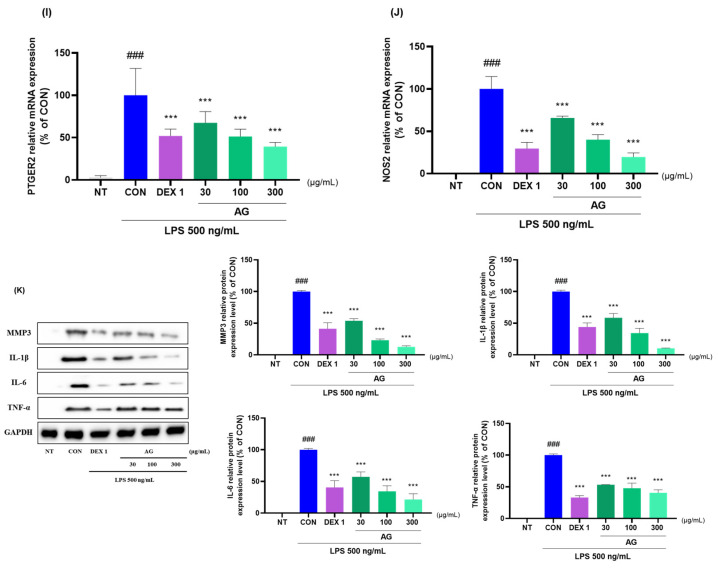
Changes in cytokine levels in the LPS-treated RAW264.7 cells. (**A**–**J**) mRNA levels of MMP-1, MMP-3, MMP-8, MMP-13, IL-1β, IL-6, TNF-α, COX-2, PTGER2, and NOS2; (**K**) protein levels of MMP-3, IL-1β, IL-6, and TNF-α. The cells were treated with DEX 1, AG 30, 100, AG 300, and LPS for 24 h. ### *p* < 0.001 vs. NT, *** *p* < 0.001 vs. CON using one-way ANOVA and Dunnett’s test.

AG and DEX 1 treatments reduced matrix metalloproteinase (MMP)-1, MMP-3, MMP-8, MMP-13, IL-1β, IL-6, TNF-α, COX-2, PTGER2, and NOS2 mRNA levels (Figure 7A–J). AG in LPS-treated RAW264.7 cells decreased the protein levels of inflammatory cytokines and mediators (Figure 7K). Furthermore, AG reduced the expression of MMP-3, IL-1β, IL-6, and TNF-α, as observed using western blot analysis. Moreover, AG exhibited anti-inflammatory effects comparable to those of DEX 1 against all cytokines.

### 3.8. Effects on Cytokine Levels in Joint Cartilage

**Figure 8:**
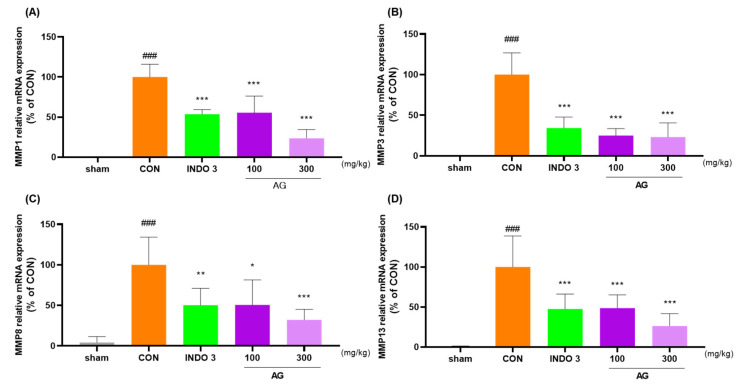

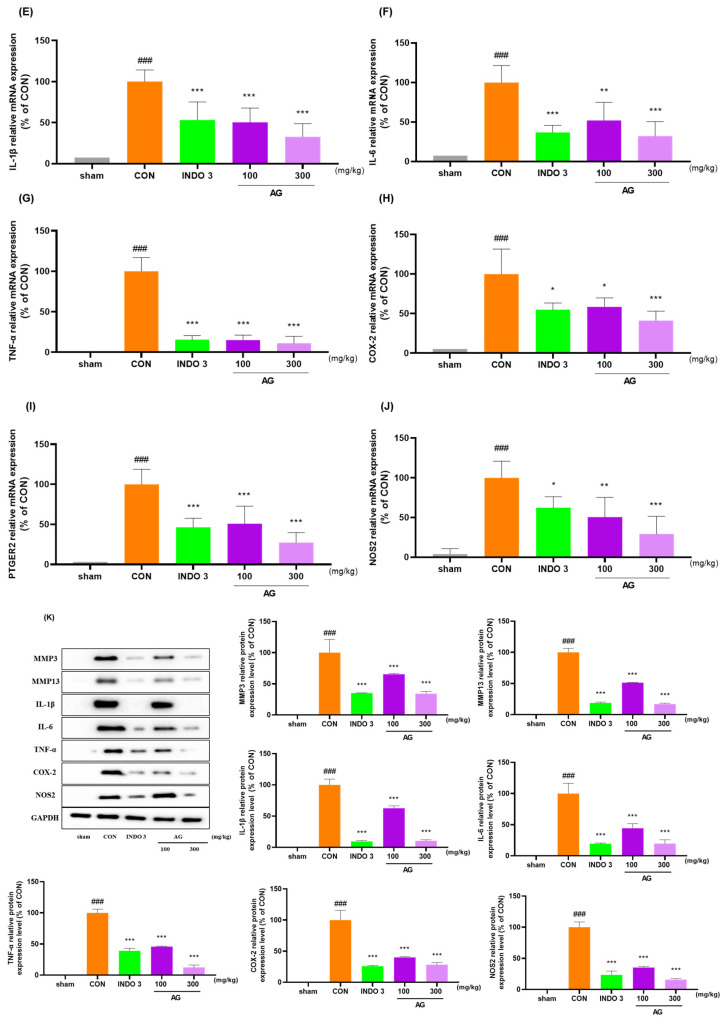
AG treatment reduced cytokine levels in the joint cartilage. (**A**–**J**) mRNA levels of MMP-1, MMP-3, MMP-8, MMP-13, IL-1β, IL-6, TNF-α, COX-2, PTGER2, and NOS2 determined using quantitative real-time polymerase chain reaction; (**K**) protein levels of MMP-3, MMP-13, IL-1β, IL-6, TNF-α, COX-2, and NOS2 determined using western blot analysis. ### *p* < 0.001 vs. sham, * *p* < 0.05 vs. CON, ** *p* < 0.01 vs. CON, *** *p* < 0.001 vs. CON using one-way ANOVA and Dunnett’s test.

AG significantly reduced the mRNA and protein levels of MMP-1, MMP-3, MMP-8, MMP-13, IL-1β, IL-6, TNF-α, COX-2, PTGER2, and NOS2 in joint cartilage compared with the levels in the CON rats (Figure 8A–J). The downregulating effect of AG on MMP-3, MMP-13, IL-1β, IL-6, TNF-α, COX-2, and NOS2 in OA rats was determined using western blot analysis (Figure 8K).

## 4. Discussion

In the present study, in vivo and in vitro models were used to observe the modulatory effects of AG on several OA pathological markers. Both MIA-induced OA rat models and acetic acid-induced writhing mouse models demonstrated superior analgesic and chondroprotective effects and functional improvement following AG treatment in a dose-dependent and consistent manner compared with the rats treated with NSAID as positive controls. Simultaneously, AG treatment exerted significant anti-inflammatory effects by inhibiting the mRNA expression of several markers in RAW264.7 cells, with almost completely consistent results in terms of cytokine inhibition in the articular cartilage. To our knowledge, this is the first study to explore the potential of AG as a DMOAD candidate for a broad range of OA-related targets. The validity of the results observed in the present study is supported by several previous studies.

In the present study, decursin and decursinol angelates were hypothesized to be the major components of AG. This hypothesis was confirmed using HPLC-UV analysis, which demonstrated the presence of these components in the AG sample. Numerous previous studies have reported the wide pharmacological potential of decursin. A study in 2021 reported that decursin could inhibit OA exacerbation by inhibiting the NF-kB and PI3K–Akt signaling pathways [26]. The preceding study demonstrated that decursin effectively inhibited IL-1β-mediated chondrocyte inflammation, exerting its effects by targeting pro-inflammatory cytokines such as IL-6, COX-2, and TNF-α. Furthermore, decursin has been reported to impede the progressive pathology of OA, including the collapse of the extracellular matrix, through its ability to modulate MMPs. Notably, these anti-inflammatory and joint destruction inhibitory effects were also consistently observed in our in vivo models, thus providing direct support for the overall anti-OA efficacy of the AG. Furthermore, decursin may inhibit osteoporosis by inhibiting receptor activator of nuclear factor kappa B (NF-κB) ligand-induced osteoclasts [27]. These findings indicate that an important therapeutic target of DMOADs is the inhibition of subchondral bone destruction that accompanies OA. Nevertheless, the OA-related indications of AG require further investigation. Additionally, our results indicate that AG may impede the advancement of numerous pathological processes by modulating multi-target inflammation in OA. Moreover, mechanisms underlying the inhibition of inflammatory pathologies by decursin and decursinol angelates in various diseases, including several types of malignancies, rheumatoid arthritis, liver fibrosis, Alzheimer’s disease, and diabetic retinopathy, are well-documented [28]. Moreover, decursinol angelate impedes the activity of MAPK and NF-κB signaling pathways, as well as LPS-treated macrophage polarization and the ensuing inflammatory response [29]. Therefore, decursinol angelate may be effective in addressing immune-mediated inflammatory diseases through a mechanism distinct from that of decursin. Moreover, the efficacy, safety, and pharmacokinetic mechanisms of both compounds in rodents have been well reproduced in humans [30]. The anti-osteoarthritic activity of AG observed in this study aligns with existing literature and is primarily attributed to its principal bioactive compounds, decursin and decursinol, as identified using HPLC. Notably, the efficacy of AG as a whole extract is evident from these positive outcomes. Diverse bioactive constituents of AG exert pleiotropic effects in various pathological conditions. Therefore, elucidating its potential as a DMOAD necessitates further investigation into its multi-target modulation and synergistic interactions from a systems biology perspective rather than focusing solely on the mechanisms of individual active components.

This study used two established in vivo models: MIA-induced OA rats and acetic acid-induced writhing mice. Our results corroborate the modulatory effects of AG on pain, joint degradation, and functional decline in OA, consistent with previous findings demonstrating its analgesic properties [24]. The chondroprotective effects of AG may be attributed to its dose-dependent, potent anti-inflammatory action on IL-1β and TNF-α in MIA rats. IL-1β regulates a complex network of chemokines, cytokines, and proteolytic enzymes in OA pathology [31]. TNF-α, a pivotal inflammatory cytokine, contributes to chondrocyte apoptosis and OA pathogenesis through cellular oxidative stress and mitochondrial dysfunction [32]. The broad anti-OA effects of AG observed in this study, compared with the active control, may be directly associated with significant inhibition of TNF-α and IL-1β in serum, the key therapeutic targets in OA inflammatory pathology. These findings indicate the potential of AG to inhibit OA pathology through its anti-inflammatory and analgesic effects. However, further investigations are warranted to elucidate its potential as a DMOAD candidate, specifically focusing on its efficacy in mitigating subchondral bone destruction and elucidating key multi-component, multi-target interactions.

The in vitro experiments in this study demonstrated that AG comprehensively modulates multiple targets involved in systemic low-grade inflammation, which is a key contributor to OA pathogenesis and progression. Beyond robust modulation of IL-1β and TNF-α, AG significantly affected MMPs, including MMP-1, MMP-3, MMP-8, and MMP-13, as well as COX-2, PTGER2, and NOS2, in vivo. These dose-dependent effects exhibited comparative advantages over NSAIDs and dexamethasone in terms of anti-inflammatory effects. Notably, these results were consistent across two in vitro models: LPS-treated RAW264.7 cells and joint cartilage tissue. Chronic inflammation in OA affects the joints, sensitizing the peripheral and central nerves and leading to mechanical allodynia and hyperalgesia [33,34]. The pronounced analgesic and functional improvements observed in both in vivo models can be attributed to the potent anti-inflammatory effects of AG against multiple targets in vitro. A notable aspect of this study was the impact of AG on MMPs, including MMP-1, MMP-3, MMP-8, and MMP-13, which are key targets in inflammatory arthritis, including OA and rheumatoid arthritis [35]. MMPs are directly involved in the progressive cartilage degradation in OA through interactions and cytokine-stimulated activation, making them essential targets for the development of DMOADs. Recent studies have confirmed a direct correlation between cellular senescence and OA pathology. Aging promotes the development of an age-related secretory phenotype characterized by an increased production of inflammatory cytokines and MMPs [36]. Consequently, MMPs are increasingly recognized as crucial therapeutic targets for DMOAD development, including novel approaches such as senolytics. In summary, the results of the in vivo and in vitro experiments in this study suggest that AG may potentially delay or mitigate the multifaceted pathological progression of OA via the potent modulation of anti-inflammatory targets through multiple bioactive components.

This screening study explored AG as a potential DMOAD candidate. However, this study has some limitations. First, we could not characterize the specific bioactive compounds and their roles underlying the effects observed in this study. Although HPLC identified two active compounds, the effects cannot be attributed solely to these compounds. Therefore, future studies should employ bioinformatics techniques to elucidate the multi-component, multi-target relationships underlying AG efficacy. Second, as a screening study, cell signaling pathways contributing to the OA modulatory effects of AG could not be identified, and this aspect requires further investigation. Third, to confirm the potential of AG as a DMOAD candidate, its ability to inhibit subchondral bone destruction and osteoporotic joint changes should be demonstrated, in addition to the observed improvements in OA inflammation, cartilage degradation, and pain. Notably, these aspects were beyond the scope of the current study but will merit future research. Finally, although the effects of AG were dose dependent, we did not establish an optimal dosage. AG has a wide range of human applications with minimal toxicity and safety concerns. However, considering the chronic nature of OA, which requires long-term administration, separate studies focusing on toxicity and safety are warranted.

## 5. Conclusions

This study demonstrates the multifaceted effects of AG on analgesia, functional improvement, and chondroprotection in animal models of OA, highlighting its potent anti-inflammatory activity against a broad spectrum of targets. These effects were consistently observed across multiple in vivo and in vitro models, showing significance relative to active controls. Furthermore, anti-inflammatory activity was observed against targets previously implicated in the inflammatory pathogenesis of OA. Our findings suggest that AG is a promising DMOAD candidate, a novel drug class that potentially inhibits the progressive pathology of OA. Nevertheless, a more comprehensive understanding of the active ingredients and their underlying mechanisms of action, as well as their effects on a wider range of OA pathologies in multiple experiments, is crucial to substantiate this hypothesis. Such investigations could further validate the potential of AG as a DMOAD candidate and provide a strong foundation for its application in OA treatment.

## Figures and Tables

**Table 1:** Condition for HPLC analysis.

	Condition
Colum	Luna C18 column (5 μm, 250 mm × 4.6 mm; Phenomenex, Torrance, CA, USA)
Mobile phase	(A) DW, (B) acetonitrile
Flow rate	0–5 min, 35–35%; 5–10 min, 35–50%; 10–30 min, 50–55%; 30–40 min, 55–70%; 40–45 min, 70–35% (B)
Injection volume	1.0 mL/min
Detection wavelength	330 nm
Temperature	30 °C

**Table 2:** MIA-induced OA model design.

Group Name	OA Model (50 μL, Intra-Articular; mg/mL)	Diet (AIN-93G)	Final Concentration (mg/kg)
sham	Saline	-	-
CON	MIA 40	-	-
INDO 3	MIA 40	+0.003% indomethacin	indomethacin 3
AG 100	MIA 40	+0.11% AG	AG 100
AG 300	MIA 40	+0.33% AG	AG 300

**Table 3:** Macroscopic score of cartilage degradation.

Grade	Cartilage Appearance
0	A typical representation of the cartilage surface
1	Modest yellowish discoloration or modest fibrillation
2	The middle or superficial layers of cartilage are affected by erosion
3	Severe deterioration that reaches the subchondral bone
4	Large-scale erosion and extensive exposure of subchondral bone

**Table 4:** mRNA primer sequences for OA rat cartilage tissues.

MMP-1	F	AAC TTG GGT GAA GAC GTC CA
	R	TCC TGT CAC TTT CAG CCC AA
MMP-3	F	GTA CGG CTG TGT GCT CAT CC
	R	TCA GCC CAA GGA ACT TCT GC
MMP-8	F	TCT GTT CTT CTT CCA CAC ACA G
	R	GCA ATC ATA GTG GCA TTC CT
MMP-13	F	ACC TTC TTC TTG TTG AG TTG GA
	R	CTG CAT TTC TCG GAG TCT A
IL-1β	F	AAC TCA ACT GTG AAA TAG CAG C
	R	TCC ACA GCC ACA ATG AGT G
IL-6	F	TCC GCA AGA GAC TTC CAG C
	R	CCT CCG ACT TGT GAA GTG G
TNF-α	F	GCA TGA TCC GAG ATG TGG AA
	R	GAT GAG AGG GAG CCC ATT TG
COX-2	F	GTT CCA ACC CAT GTC AAA AC
	R	TGT CAG GAA TCT CGG CGT AG
Ptger2	F	TGT GTG TAC TGT CCG TCT GC
	R	CAG GGA TCC AGT CTC GGT GT
NOS2	F	AGT CAA CTA CAA GCC CCA CG
	R	GCA GCT TGT CCA GGG ATT CT
GAPDH	F	CTT GTG ACA AAG TGG ACA TTG TT
	R	TGA CCA GCT TCC CAT TCT C

COX: cyclooxygenase; GAPDH: Glyceraldehyde 3-phosphate dehydrogenase; IL: interleukin; MMP: matrix metalloproteinase; Ptger2: prostaglandin E receptor 2; NOS: nitric oxide synthase; TNF: tumor necrosis factor.

**Table 5:** mRNA primer sequences for LPS-treated RAW264.7 cells.

MMP-1	F	ATG CCT AGC CTT CCT TTG CT
	R	TTC CAG GTA TTT CCA GAC TG
MMP-3	F	AAG TTC CTC GGG TTG GAG AT
	R	ACC AAC ATC AGG AAC ACC AC
MMP-8	F	CAA TCA ATT CCG GTC TTC GA
	R	GGT TAG CAA GAA ATC ACC AGA
MMP-13	F	AAC CAA GAT GTG GAG TGC CT
	R	GAC CAG ACC TTG AAG GCT TT
IL-1β	F	CCA GCT TCA AAT CTC GCA GC
	R	GTG CTC ATG TCC TCA TCC TGG
IL-6	F	CAC TTC ACA AGT CGG AGG CT
	R	CAA GTG CAT CAT CGT TGT TC
TNF-α	F	GAG AAG TTC CCA AAT GGC CT
	R	AGC CAC TCC AGC TGC TCC T
COX-2	F	ATC CAT GTC AAA ACC GTG GG
	R	TTG GGG TGG GCT TCA GCA G
Ptger2	F	CTG GTA ACG GAA TTG GTG C
	R	TGG CCA GAC TAA AGA AGG TC
NOS2	F	ACC AAG ATG GCC TGG AGG AA
	R	CCG ACC TGA TGT TGC CAT TG
GAPDH	F	ATG GTG AAG GTC GGT GTG
	R	GCC GTG AGT GGA GTC ATA C

COX: cyclooxygenase; GAPDH: Glyceraldehyde 3-phosphate dehydrogenase; IL: interleukin; MMP: matrix metalloproteinase; NOS: nitric oxide synthase; Ptger2: prostaglandin E receptor 2; TNF: tumor necrosis factor.

## Data Availability

All data from this study are included in the main body of the article.
